# Amino Acid-Mediated Metabolism: A New Power to Influence Properties of Stem Cells

**DOI:** 10.1155/2019/6919463

**Published:** 2019-12-05

**Authors:** Jilan Liu, Xianyun Qin, Dongfeng Pan, Bin Zhang, Feng Jin

**Affiliations:** ^1^Department of Central Laboratory, Affiliated Hospital of Jining Medical University, Jining Medical University, Jining, Shandong 272029, China; ^2^Department of Radiology and Medical Imaging, University of Virginia, Charlottesville, VA 22908, USA; ^3^Department of Laboratory Medicine, Affiliated Hospital of Jining Medical University, Jining Medical University, Jining, Shandong 272029, China; ^4^Department of Neurosurgery, Affiliated Hospital of Jining Medical University & Shandong Provincial Key Laboratory of Stem Cells and Neuro-Oncology, Jining, Shandong 272029, China

## Abstract

The self-renewal and differentiation potentials of stem cells are dependent on amino acid (AA) metabolism. We review the literature on the metabolic preference of both cancer and noncancer stem cells. The balance in AA metabolism is responsible for maintaining the functionality of noncancer stem cells, and altering the levels of AAs can influence the malignant biological behavior of cancer stem cells. AAs are considered nutrients participating in metabolism and playing a critical role in maintaining the activity of normal stem cells and the effect of therapy of cancer stem cells. Targeting AA metabolism helps inhibit the stemness of cancer stem cells and remodels the function of normal stem cells. This review summarizes the metabolic characteristics and regulation pathways of AA in different stem cells, not only from the nutritional perspective but also from the genomic perspective that have been reported in the recent five years. In addition, we briefly survey new therapeutic modalities that may help eradicate cancer stem cells by exploiting nutrient deprivation. Understanding AA uptake characteristics helps researchers define the preference for AA in different stem cells and enables clinicians make timely interventions to specifically target the cell behavior.

## 1. Introduction

Stem cells are poorly differentiated cells with self-renewal ability and can be divided into cancer stem cells (CSCs) and normal stem cells based on their cell proliferation ability and into pluripotent, multipotent, and monopotent stem cells based on their differentiation potential. Pluripotent stem cells, such as embryonic stem cells (ESCs), differentiate into various types of tissue cells, and the stability of this differentiation process maintains the normal growth and development of the human body. CSCs have unlimited proliferation capacity and are closely related to the recurrence, metastasis, and drug resistance in tumors; few CSCs induce tumor occurrence [1, 2].

Because they have high heterogeneity, eliminating CSCs may represent a permanent cure for cancer [3–5]. Tumor tissues include endothelial cells, stromal fibroblasts, immune cells, and malignant cancer cells; the cadres of these cells constitute the tumor microenvironment (TME). Cancer cells encounter numerous challenges and thus readjust their metabolic properties in their TMEs [6]. A complex TME provides a unique niche to CSCs. Accumulating evidence suggests that the normal stem cell niche is altered in patients with hematological neoplasms and that the “neoplastic niche” promotes malignancy and suppresses normal blood cell development in such patients [7]. CSCs alter the TME by transforming adjacent fibroblasts into cancer-associated fibroblasts (CAF), and CAFs can activate CSC growth by metabolites (such as lactic acid, ketone bodies, and glutamine) [8–10]. Hypoxia and nutrient deprivation result in a buildup of lactic acid, acidifying the TME; this protects CSCs from immune recognition [11, 12]. Under chronic acidosis conditions, tumors prioritize glutamine intake [13]. Under hypoxic conditions, tumor cells strongly express hypoxia-inducible factor 1*α* (HIF-1*α*) and return to a stem-like phenotype through dedifferentiation [14, 15]. When cultured under hypoxic conditions, induced pluripotent stem cells (iPSCs) change their gene expression to resemble the phenotype of CSCs; this provides added support to this theory [16]. Hypoxia promotes cell survival and induces the epithelial-mesenchymal transition (EMT) [17]. EMT-related factors, including HIF-1*α*, WNT, and Snail, regulate cellular metabolism, and the EMT-related metabolites glutamine, glutamate, and alanine and high lactate concentration are associated with poor survival and high metastatic potential in patients with breast cancer [18, 19]. The intraniche metabolic crosstalk contributes to the production of an adaptive phenotype of tumor cells [6]. Therefore, it is crucial to explain the metabolic characteristics in different stem cell niches.

We focus on the importance of amino acid (AA) metabolism to the properties of stem cells. This review elaborates the function of AA metabolism in the CSCs and normal stem cells. Defining the metabolic characteristics of different types of cells may increase the specificity of cancer treatments.

### 1.1. AA Metabolism of CSCs

The status of cancer cells can be reprogrammed through metabolic remodeling while their dedifferentiation ability for the induced stem-like phenotype is maintained [20–22]. This capability of switching between differentiated somatic and stem cell states is called cell plasticity [23]. When performing tumor therapy, both genomic instability and microenvironment-driven selection support tumor heterogeneity and enable the development of resistant cells with stem-like properties because of cell plasticity [24]. Some cellular signals, such as WNT/*β*-catenin, NOTCH, NF-*κ*B, and JAK/STAT, facilitate such phenotypic plasticity. Some cytokines, such as the vascular endothelial growth factor and epidermal growth factor, are positively correlated with the reprogramming of stem cell properties [25–27]. The behavior of CSCs somewhat depends on niche stability; hypoxia and acidosis lead to a change of niche in the TME [15, 24, 28]. The extracellular matrix is a dynamic TME in the stem cell niche, which is regulated by components, such as nutrients and molecules. An unstable extracellular matrix leads to abnormal behavior of stem cells [28–30]. In addition to hypoxia and acidosis, AAs are main contributors to cell survival in a niche, particularly that of CSCs. Many studies have recently demonstrated that AAs regulate cancer cell function (AAs) [19–21]. Extracellular free AAs affect the malignant biological behavior of tumor cells through cell metabolism, and understanding the metabolic characteristics of CSCs can help researchers seeking to eliminate them.

#### 1.1.1. AA Metabolism and CSC-Related Phenotypic Properties

**Figure 1:**
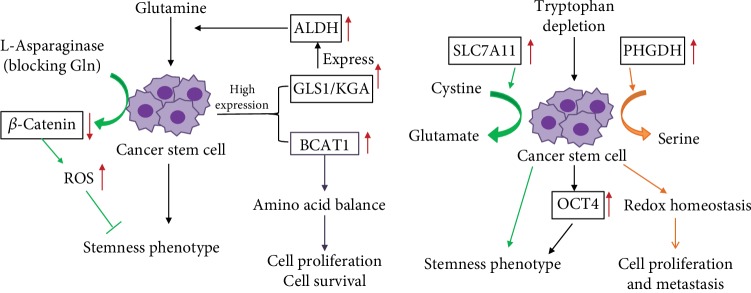
Effect of amino acids on the properties of cancer stem cells (CSCs).

Glutamine is the most abundant and widely used AA in the human body. The body synthesizes glutamine itself, and glutamine is thus called a nonessential AA. It is hydrolyzed into glutamic acid, aspartic acid, and other metabolites by glutaminase. Since the discovery of tumor metabolomics, increasing evidence has suggested that tumor cell growth is highly dependent on glutamine [31–33]. Whether this dependence derives from stem cells in tumor cells is unclear, so scholars have described the regulation of glutamine metabolism in CSCs. Mani et al. [34] demonstrated that the epithelial-mesenchymal transition (EMT) confers tumor cells with self-renewal ability and promotes CSC production. So, we question whether there is a difference in amino acid metabolism between EMT-related epithelial cell carcinoma and cancer stem cells. Aguilar et al. [35] performed a metabolomic comparison of two prostate cancer cell lines—PC-3M and PC-3S. PC-3M was rich in stemness phenotype, but PC-3S was not rich. They discovered that PC-3M has higher levels of glutamine metabolism and higher expression of glutaminase than PC-3S. The Warburg effect is strong in stem cells, and the strength of the Warburg effect is negatively related to the degree of cell differentiation [36]. Elevated glutamine metabolism can impair the damage caused by acidic substances produced by the Warburg effect, and this protection is achieved through glutathione synthesis, NADPH production, and pH homeostasis [37, 38]. Although glutamine plays a critical role in various tumor tissues [39, 40], the properties of tumor stem cells in various types of tissue remain to be explored. Tardito et al. [41] demonstrated that glioma stem cell proliferation is inhibited in the absence of glutamine, whereas increasing the activity of glutamine synthetase enables tumor stem cells to grow under glutamine starvation. Through in vivo and in vitro experiments, Kamarajan et al. [42] demonstrated that the expression of glutaminase 1 (GLS1) and acetaldehyde dehydrogenase (ALDH) in CSCs of primary and metastatic head and neck cancer tissues was high. GLS1 is a GLS isoenzyme, of which there are two subtypes—kidney glutaminase (KGA) and glutaminase C (GAC). These subtypes are highly expressed in different cancers, and targeting glutaminase 1 (both KGA and GAC) was discovered to reduce the stemness phenotype in vitro and tumorigenicity in vivo through the reactive oxygen species (ROS)/Wnt/*β*-catenin signaling pathway [43–45]. Accumulating evidence has been obtained that ALDH helps maintain stem cell properties, and ALDH is also regarded a stem cell marker named the stemness marker [46]. Therefore, glutaminase maintains the in vitro CSC stemness phenotype and tumorigenesis in vivo. Head and neck CSCs (CD44 high/ALDH high) have higher glutaminase and glutamate levels than CD44 low/ALDH low cells and are more likely to be spherical. Additionally, glutamine directs CD44 low/ALDH low cells into stemness cells. Glutaminase promotes transcription and translation of ALDH expression, and glutaminolysis regulates tumorigenesis and CSC metabolism by regulating ALDH expression. These findings indicate that glutamate is a potential marker of cancer metabolism and provides a new theoretical basis for tumor diagnosis. On the basis of this, Liao et al. [47] further demonstrated that glutamine maintains the mechanism of stem cell stemness and used L-asparaginase to mimic the state of intracellular glutamine withdrawal. They discovered that glutamine deprivation increases intracellular ROS levels and downregulates the *β*-catenin signaling pathway. The above content (see Figure 1) illustrates the effect of glutamine on CSCs. Recently, cystine/glutamate antiporter xCT become a hot spot for researchers; SLC7A11 encodes the xCT. Polewski et al. [48] confirmed that the cystine/glutamate antiporter system (SLC7A11) is upregulated in glioma, and the overexpression of SLC7A11 enhances the stemness phenotype of glioma stem cells (see Figure 1). Targeting of SLC7A11 combined with chemotherapy drugs can reduce the likelihood of cancer resistance and recurrence and improve the survival of patients with glioblastoma (GBM). Kim et al. [49] revealed a new mechanism in which metformin inhibits CSCs through the glutamine metabolic pathway. Kim et al. used two types of colon cancer cells (SW620 and HT29) as an experimental model, and SW620 was discovered to be resistant to metformin, whereas HT29 was sensitive to metformin. Studies have confirmed that ASCT2 is more highly expressed in SW620-derived CSCs compared with HT29, and knocking out ASCT2 or inhibiting glutamine can enhance the inhibitory effect of metformin on CSCs (see Figure 1). The resistance of metformin can thus be overcome by inhibiting the glutamine metabolism pathway. Wang et al. [40] demonstrated that glutamine transporter 2 (ASCT2) is highly expressed in prostate cancer tissues, and inhibiting the expression of ASCT2 in prostate cancer can reduce glutamine uptake, leading to downregulation of E2F cell cycle pathway proteins and mTORC1 pathway inhibition, thereby inhibiting the growth of prostate cancer cells. Therefore, we question whether the high expression of ASCT2 is closely related to prostate CSCs and aim to obtain a novel concept for prostate CSC metabolism therapy. In recent years, branched-chain AA aminotransferase 1 (BCAT1) was discovered to be involved in the progression of glioma, ovarian cancer, liver cancer, breast cancer, and leukemia [50–54], but less in CSCs. Raffel et al. [55] performed proteomic analysis of stem cells and nonstem cells in acute myeloid leukemia (AML) and found that BCAT1 was significantly highly expressed in stem cells (see Figure 1). Targeting BCAT1 will become a new target for stem cell therapy in leukemia.

The presented literature review indicates the necessary effects of the presence of glutamine on stem cell characteristics. Regarding other AAs, phosphoglycerate dehydrogenase (PHGDH) is a metabolic enzyme used in serine synthesis, and overexpression of PHGDH has been associated with mortality of patients with breast cancer [56]. In recent years, researchers have demonstrated that high PHGDH expression in breast CSCs causes maintained cell proliferation and metastasis ability through the maintenance of redox homeostasis [57]. Samanta and Semenza [58] showed that PHGDH reprograms cell metabolism toward increased glycolysis and suppressed oxidative phosphorylation. A more active serine synthesis pathway helps hypoxic tumor stem cells adapt to hypoxia for cell survival. The kynurenine (Kyn) pathway is the main direction of tryptophan metabolism and the crucial mechanism of immune escape; the immunosuppressive effect of the Kyn pathway has been attributed to mainly reduced tryptophan levels [59]. Two main rate-limiting enzymes of tryptophan metabolism, indoleamine-2,3-dioxygenase (IDO) and tryptophan-2,3-dioxygenase, are strongly expressed in tumors and are correlated with poor prognosis of patients with cancer [60–63]. An abnormal increase in tryptophan metabolism enzyme activity leads to depletion of tryptophan levels in the TME. Recent research has demonstrated that tryptophan depletion and hypoxia preserve the phenotype of CSCs by inducing the enhancement of OCT4 transcription; therefore, various tryptophan derivatives can be used to inhibit CSCs [64] (see Figure 1).

Glutamine promoted CSCs' proliferation and maintained stemness phenotype. Glutaminase 1 (GLS1) was highly expressed in CSCs and promoted the expression of acetaldehyde dehydrogenase (ALDH); its overexpression induced glutamine metabolism. BCAT1 promoted HSC proliferation and survival via maintenance of amino acid balance. SLC7A11, which function as cystine/glutamate antiporter, was also highly expressed and enhances the stemness phenotype of glioma stem cells (GSCs). Phosphoglycerate dehydrogenase (PHGDH) promoted serine synthesis and maintained cell proliferation and metastasis ability via the maintenance of redox homeostasis. Tryptophan depletion preserved the CSC phenotype via inducing the enhancement of OCT4 transcription.

#### 1.1.2. AA Metabolism and CSC-Related New Therapeutic Modalities

**Figure 2:**
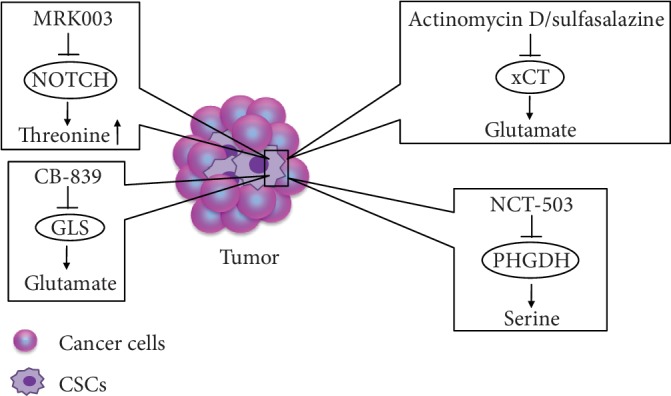
New therapeutic agents targeting AA metabolism.

Metabolic drugs are used to treat tumors on the basis of differences in the expression of substances in tumor cells and normal tissues; they mainly target a specific AA or a key metabolic enzyme for dietary or drug intervention. With increasing numbers of studies investigating the effects of AA metabolism on the regulation of tumor stem cell phenotypes, targeting AA metabolism pathways will become a popular topic in cancer therapy research. Although AA metabolism pathways have a strong impact on the properties of CSCs, limited studies have evaluated the treatment of tumors by targeting AA metabolism pathways. Here, we summarize some potential therapeutic modalities (Figure 2).

Targeting the function of membrane transporters in CSCs is a potential new therapeutic strategy. In vivo and in vitro experiments have demonstrated that pharmacological deletion of the cystine/glutamate transporter xCT causes AA starvation in estrogen receptor-positive breast cancer cells and suppresses the proliferation of these cells [65]. Actinomycin D has been identified as a potential antitumor agent that significantly inhibits activity of liver CSCs without affecting normal hepatocytes; the inhibitory effect on CSCs results from the inhibition of xCT expression and CD133 synthesis [66]. In addition, the xCT inhibitor, sulfasalazine, leads to the impairment of glutathione synthesis and induces ROS generation, thereby triggering oxidative damage in head and neck squamous cell carcinoma [67]. Tumor cells have strong drug resistance, and new drugs such as small molecule inhibitors are urgently needed for treating tumors. NCT-503, a molecular inhibitor targeting PHGDH, exerts an antitumor effect by enhancing the chemotherapeutic sensitivity of erlotinib in lung cancer [68]. CB-839, GLS inhibitor, inhibits the growth of AML cells by reducing the rate of conversion of Gln to glutamate [69]. Notch signaling promotes tumorigenesis and also plays a crucial role in stem-cell-like cells [70, 71]. Notably, Kahlert et al. [72] demonstrated that the Notch inhibitor MRK003 has different roles in GBM cells and U87NS glioma stem cell (GSC) spheres. In GBM cells, glutamate and glutamine levels are decreased, but in U87NS GSCs, threonine and lactate levels are significantly increased. Literature does not mention how threonine metabolism regulates tumor stem cell functionality. The aforementioned agents may become effective antitumor drugs that target the AA metabolism pathways of CSCs. In addition, many studies have focused on IDO inhibitors targeting tumors, but few studies have explored whether the antitumor effect of IDO inhibitors originates from anti-CSCs.

Actinomycin D (ActD), sulfasalazine target xCT, and CB-839 target GLS to reduce glutamate synthesis. NCT-503 targets PHGDH to reduce serine synthesis. MRK003 targets Notch to induce the threonine level.

### 1.2. AA Metabolism of Normal Stem Cells

#### 1.2.1. Embryonic Stem Cells and Induced Pluripotent Stem Cells

ESCs are typical pluripotent stem cells with the potential to form complete individuals. Studying the metabolic characteristics of ESCs has been critical to the development of regenerative medicine. Due to ethical constraints, few studies have investigated human ESCs (hESCs); instead, mouse ESCs (mESCs) or induced pluripotent stem cells (iPSCs) have been employed. iPSCs are of a similar type to ESCs. In 2006, Takahashi and Yamanaka [73] used viral vectors to transfer four transcription factors (OCT4, SOX2, KLF4, and C-MYC) to receptor cells to maintain the stemness phenotype in a process called cell reprogramming. Due to the similar functions of iPSCs and ESCs, numerous researchers have circumvented the ethical controversy around ESCs by studying iPSCs instead. However, in research on iPSCs, there is a risk of a low induction rate and carcinogenesis.

**Figure 3:**
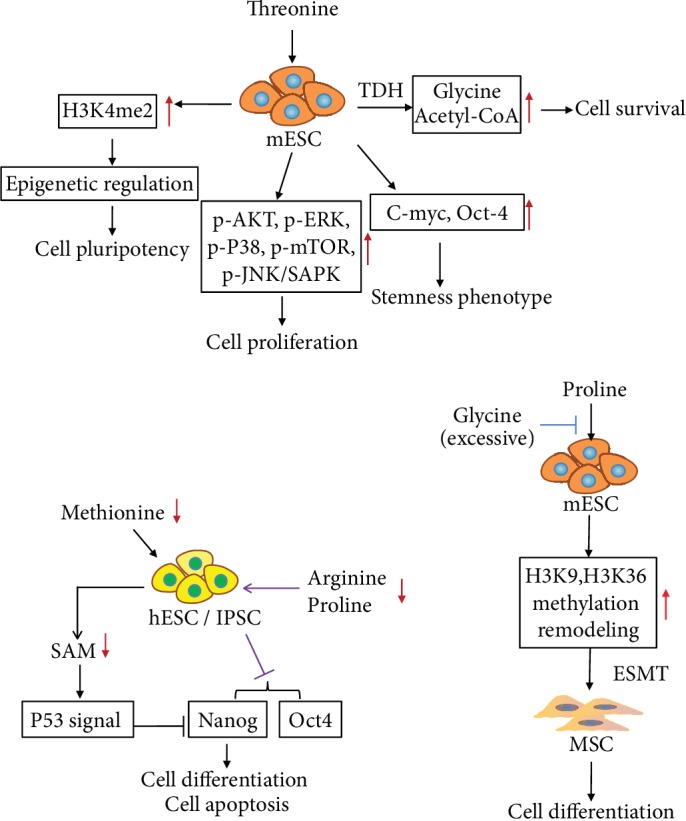
Effect of amino acids on the properties of embryonic stem cells (ESCs).

In recent years, researchers have discovered that some AAs are highly sensitive to maintaining stem cell pluripotency and differentiation ability. Therefore, the AA metabolism characteristics of stem cells may be used to improve the induction rate of iPSCs and application security. Since 1998, researchers have found that the expression of stem cell marker (c-myc, oct4) is reduced when cells are cultured in Dulbecco's modified Eagle medium that lacks threonine. Subsequently, the impact of AA metabolism regulation on embryonic development was discovered [74]. When threonine was resupplied, the proliferative capacity of mESCs was restored by activating AKT, ERK, P38, JNK/SAPK, and mTOR [75, 76]. In a recent study, Chen and Wang [77] demonstrated that threonine is involved in the maintenance of epigenetic regulation of mESC pluripotency. Threonine dehydrogenase (TDH), which converts threonine into glycine (for single carbon metabolism) and acetyl-CoA (for energy production), is significantly necessary to the survival of the mESCs. Threonine depletion and TDH inhibition result in significantly decreased levels of H3K4me2. Demethylation of histone H3 at lysine 4 (H3K4me3) is the result of consuming cellular S-adenosyl methionine (SAM). H3K4me3 is dependent on the consumption of cellular SAM. Thus, threonine and methionine, used as donor molecules for histone methylation, are essential to the growth and differentiation of hESCs. Furthermore, based on this study [77], Shyh-Chang et al. [78] discovered that placing glycine and pyruvate in the medium prevented the threonine-starvation-induced death of mESCs (see Figure 3), illustrating the threonine metabolism of ESCs. Shiraki and Kume reported that hESCs and iPSCs require large amounts of methionine (Met) and express high levels of Met-metabolizing enzymes. Met deprivation leads to a rapid decrease in intracellular SAM and SAM-regulated gene expression through supporting methyls for DNA and histone. The reduction of SAM triggers p53 signaling, reduces the expression of the pluripotency marker Nanog, and differentiates hESCs and iPSCs into three germ layers [79]. However, when deprived of Met for a long period, cells move toward apoptosis (see Figure 3).

Kilberg et al. [75] comprehensively demonstrated that the self-renewal and differentiation of mESCs are dependent on proline metabolism, and excessive glycine levels can block proline-induced stem cell differentiation. The researchers also discovered that L-proline can induce ESC transformation into mesenchymal stem cells while genome remodeling with H3K9 and H3K36 methylation occurred [80]. He and others enriched the regulation of AA in ESCs [81], confirmed that arginine and proline depletion can impair the pluripotency of hESCs, and indicated that the downregulation of pluripotency is due to the reduction of Nanog and Oct4 expression (see Figure 3), illustrating the function of proline, arginine, and glycine in mESCs. He et al. [81] reported that deprivation of glycine, serine, and tyrosine can downregulate Nanog expression on the first occasion but enhance the endodermal differentiation potential. Under the double deprivation of methionine and cysteine, the self-renewal ability of hESC H9 is reduced. These studies provide a reference for human embryo development and ESC research.

Threonine promoted cell survival via metabolism by TDH to glycine, but excessive glycine inhibited cell differentiation by blocking proline. Threonine input maintained the properties of stem cells including stemness phenotype and pluripotency via upregulating H3K4me2 and c-Myc and Oct4 expression. Threonine induced cell proliferation by activating signal pathways (AKT, ERK, P38, mTOR, and JNK). Adding proline to the medium promoted the differentiation of mouse embryonic stem cells into mesenchymal stem cells. Double depletion of arginine and proline reduced the expression of Nanog and Oct4 in human embryonic stem cells. Methionine deprivation reduced Nanog expression via activating the P53 signal pathway.

#### 1.2.2. Hematopoietic Stem Cells

Hematopoietic stem cells (HSCs) are the original cells in blood, have the potential to differentiate into various blood cells, are regarded as typical multipotent stem cells, and play a key role in maintaining the stability of blood components. At present, HSCs are mainly used to treat leukemia through bone marrow transplantation. However, such transplantation causes difficulties such as immune rejection and metabolic disorders.

**Figure 4:**
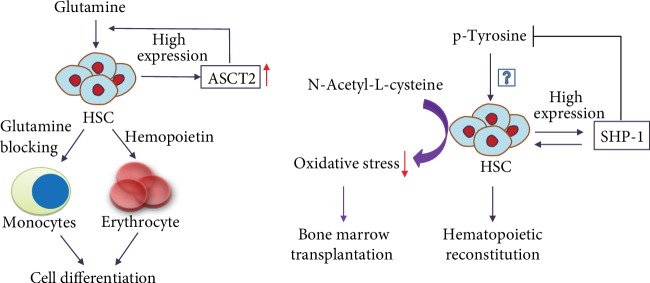
Effect of amino acids on the properties of hematopoietic stem cells (HSCs).

An imbalance of AA metabolism can affect the proliferation and survival of HSCs in vivo and be harmful to them. Next, Wilkinson et al. [82] employed metabolomics to confirm that a balance of branched-chain AAs (BCAAs) helps maintain hematopoietic stem cell proliferation and cell survival. HSCs are sensitive to BCAA levels, especially that of proline, and the balance between these levels. However, interestingly, an imbalance of BCAAs inhibits HSC growth more strongly than insufficiency of three AAs, confirming that AA balance affects HSC survival more strongly than AA levels. Therefore, the imbalance of AA may be an essential promoter of dysfunction of HSCs. Studies [82, 83] highlight a critical role for valine balance in HSC homeostasis, but the mechanism is unclear. Therefore, enhancement of nutritional AA balance can be used as a combined treatment for bone marrow transplantation. Oburoglu et al. [84] confirmed that the glutamine transporter (ASCT2) was highly expressed in HSCs. The ability of HSCs to differentiate into erythrocytes is dependent on ASCT2 and activation of glutamine metabolism. Blocking glutamine metabolism can cause HSCs to differentiate into monocytes instead (see Figure 4). SHP-1 is a class of protein tyrosine phosphatases with an SH2 domain that controls intracellular phosphorylation levels of tyrosine, and the SHP-1 proteins are expressed in all hematopoietic cells. A lack of SHP-1 weakens the ability of HSCs to self-renew, and in vivo experiments have confirmed that the HSCs in SHP-1 knockout mice have no hematopoietic reconstitution ability [85]. However, whether the phosphorylation level of tyrosine is involved in the hematopoietic reconstitution of HSCs is worth further exploration (see Figure 4). The current method used for determining the self-renewal and differentiation potentials of HSCs is HSC implantation into immunodeficient mice, but the implantation success rate is extremely low. Hu et al. [86] used the AA derivative N-acetyl-L-cysteine to reduce oxidative stress during implantation and increased the HSC implantation efficiency (see Figure 4). With our deep study of the AA metabolism of HSCs, the efficiency of bone marrow transplantation can be improved in the future through nutritional support or combined antimetabolic drugs.

HSCs highly expressed glutamine transporter (ASCT2). ASCT2 activated glutamine metabolism resulting in cell differentiation into erythrocyte. Blocking glutamine metabolism resulted in cell differentiation into monocytes. SHP-1 reduced phosphorylation of tyrosine and induced hematopoietic reconstitution ability. Inputting N-acetyl-L-cysteine promoted bone marrow transplantation via inhibiting oxidative stress.

## 2. Conclusion and Discussion

After performing synthetic analysis, we discovered that threonine, proline, and methionine affect the process through which ESCs and iPSCs differentiate into different tissues; however, HSCs and CSCs are highly sensitive to glutamine. This may be due to the preference of HSCs and CSCs to an acidic niche. An acidic environment enhances drug resistance by reducing chemotherapeutic drug permeability and promoting drug efflux [24]. In addition, hypoxia induces high glutamine metabolism. Hypoxia induces HIF-1*α* to maintain the stem-phenotype of CSCs, and the expansion of myeloid progenitors induces hypoxia due to oxygen depletion and stabilizes HIF-1*α* in the bone marrow microenvironment; thus, hypoxia-induced HIF-1*α* activation is essential to HSC mobilization [15, 87]. The diversity of AAs taken up by ESCs and iPSCs is related to the diversity of their differentiation orientation. In the future, inducing normal stem cell-oriented differentiation may be dependent on exogenous AA intervention. Tryptophan depletion induces the stemness phenotype of CSCs, which may correspond to the inhibition of the T cell response [88]. Tryptophan metabolism produces an immunosuppressive Kyn, and based on the aforementioned theories, tryptophan metabolism inhibition may enhance the tumor immune response; some relevant inhibitors are currently undergoing clinical trials [89]. However, whether or not the inhibition of tryptophan metabolism can reduce the drug resistance of CSCs requires further investigation.

Understanding the metabolic expression profiles of different tissues and organs can help researchers to achieve the objective of differentiation from stem cells into specific tissue types according to different preferences of tissues and organs for various AAs; this can be achieved by altering the nutritional input or gene regulation. Stem cells derived from different pathological types have different AA metabolism patterns, which may be related to their microenvironment and genetic background. Therefore, according to the metabolic characteristics of different CSCs, more antitumor modalities against specific CSCs can be developed. The metabolic differences between stem cell types provide a theoretical basis for developing effective antitumor drugs that do not damage normal cells [90]. Furthermore, according to the characteristics of AA metabolism in different tumors, the therapeutic effect of antitumor drugs may be improved by changing dietary habits in the future.
